# Preterm Birth and Kidney Health: From the Womb to the Rest of Life

**DOI:** 10.3390/children11101213

**Published:** 2024-10-02

**Authors:** You-Lin Tain, Chien-Ning Hsu

**Affiliations:** 1 Division of Pediatric Nephrology, Kaohsiung Chang Gung Memorial Hospital, Kaohsiung 833, Taiwan; tainyl@cgmh.org.tw; 2 College of Medicine, Chang Gung University, Taoyuan 333, Taiwan; 3 Department of Pharmacy, Kaohsiung Chang Gung Memorial Hospital, Kaohsiung 833, Taiwan; 4 School of Pharmacy, Kaohsiung Medical University, Kaohsiung 807, Taiwan

**Keywords:** nephron number, congenital anomalies of the kidney and urinary tract (CAKUT), preterm birth, chronic kidney disease, neonatal acute kidney injury, developmental origin of health and disease (DOHaD), pregnancy

## Abstract

Chronic kidney disease (CKD) is a widespread condition often resulting from multiple factors, including maternal influences. These risk factors not only heighten the likelihood of developing CKD but increase the risk of a preterm birth. Adverse events during nephrogenesis can disrupt kidney development, leading to a reduced number of nephrons. As survival rates for preterm infants improve, more individuals are living into adulthood, thereby elevating their risk of CKD later in life. This review aims to explore the connections between preterm birth, kidney development, and the increased risk of CKD, while proposing practical solutions for the future through a multidisciplinary approach. We examine human studies linking preterm birth to negative kidney outcomes, summarize animal models demonstrating kidney programming and reduced nephron numbers, and consolidate knowledge on common mechanisms driving kidney programming. Additionally, we discuss factors in the postnatal care environment that may act as secondary insults contributing to CKD risk, such as acute kidney injury (AKI), the use of nephrotoxic drugs, preterm nutrition, and catch-up growth. Finally, we outline recommendations for action, emphasizing the importance of avoiding modifiable risk factors and implementing early CKD screening for children born preterm. Together, we can ensure that advancements in kidney health keep pace with improvements in preterm care.

## 1. Introduction

In 2020, an estimated 13.4 million newborns, or roughly 1 in 10, were born preterm [1]. Over the past decade, global preterm birth rates have not reduced. Despite significant advancements in perinatal care that have greatly improved survival rates for preterm infants, prematurity continues to be the leading cause of neonatal morbidity and mortality [2]. Notably, as survival rates for preterm infants rise, so does the number of these individuals living into adulthood, which increases their risk of developing various adult diseases.

The Barker hypothesis, also known as the developmental origins of health and disease (DOHaD) hypothesis, proposes that adverse conditions during fetal or early life can have long-lasting effects on an individual’s health [3,4]. Factors contributing to a preterm birth, such as poor maternal nutrition, maternal illness, smoking, excessive alcohol consumption, medication use, maternal stress, exposure to environmental pollutants and toxins, and complications during pregnancy, are associated with these early-life risks [2]. Importantly, these same early-life risk factors are closely linked to the development of adult diseases [5]. Additionally, premature infants face a higher risk of chronic health disorders as they grow older [6]. These observations suggest that prematurity may serve as a critical factor connecting early-life adversities to the onset of adult diseases.

Up to 10% of the population worldwide is affected by chronic kidney disease (CKD) [7]. CKD can stem from various adverse conditions encountered early in life [8,9,10]. In response, World Kidney Day 2016 highlighted the importance of addressing kidney health from childhood and understanding its impact on adult CKD [11]. During fetal development, the kidney is vulnerable to adverse environments, which can lead to functional and structural changes known as kidney programming [12,13]. Since kidney development is completed by full-term birth, premature infants often experience incomplete nephrogenesis, resulting in a reduced number of nephrons [14]. Although the long-term impact of prematurity on kidney function and CKD is not fully understood [15], it is recognized that disease development is generally the result of multiple contributing factors, or “hits”, over time. CKD exemplifies this multiple-hits nature [16]. Prematurity itself is often caused by a combination of factors, and each “hit” can contribute to CKD onset. Moreover, prematurity can act as an initial “hit”, making the kidney more susceptible to subsequent postnatal hits or exacerbated conditions.

Although research into the long-term outcomes of premature infants has been growing [17,18,19], the specific impact of prematurity on kidney health remains less well understood. Emerging evidence from both basic science and clinical studies indicates that prematurity may play a significant role in kidney programming and subsequent kidney health [20,21,22,23]. This growing body of evidence suggests that premature birth can have lasting effects on kidney development, potentially predisposing individuals to kidney disease later in life. Studies have begun to shed light on how early life disruptions can influence kidney health over the long term, but many questions remain about the mechanisms involved and the extent of the impact.

This narrative review aims to comprehensively explore and evaluate the existing evidence regarding the effects of prematurity on kidney development and health. By examining both experimental findings and clinical observations, the review seeks to clarify the role of prematurity in the progression to kidney disease and to identify potential areas for future research and intervention.

## 2. Preterm Birth and Kidney Development

### 2.1. Preterm Birth

Preterm birth, or prematurity, is defined as birth occurring before 37 weeks of gestation. Infants born prematurely are categorized into distinct age groups based on their gestational age (GA) at birth [24]. These groups include extremely preterm infants (<28 weeks), very preterm infants (28–31 weeks), moderately preterm infants (32–33 weeks), and late preterm infants (34–36 weeks). While preterm birth and low birth weight (LBW, birth weight < 2500 g) are distinct conditions, they are often interconnected. Preterm birth frequently results in low birth weight, and both conditions may share similar underlying factors.

Even though much research has been carried out over the last decade, the incidence of preterm birth remains relatively unchanged [1]. The most likely explanation is that preterm birth represents a syndrome with various underlying causes that may interact synergistically to contribute to its onset [25]. Preterm birth can occur spontaneously due to preterm labor, preterm rupture of membranes (PROM) or, occasionally, cervical insufficiency [2]. However, about one-third of preterm births are medically induced when continuing the pregnancy poses risks (e.g., pre-eclampsia).

Preterm birth poses significant challenges in both pediatrics and obstetrics, being a multifactorial issue. While the exact causes of preterm labor and the PROM are often not fully understood, several risk factors have been identified. These include inadequate maternal nutrition, extreme maternal ages, pre-pregnancy body weight, pregnancy characteristics such as multiple gestations and short intervals between pregnancies, as well as abnormalities in the uterus or cervix. Additionally, a history of previous preterm deliveries, infections or inflammation, maternal lifestyle choices (like smoking, alcohol consumption, and illicit drug use), exposure to environmental chemicals, stress, underlying maternal health conditions, and certain medications can all contribute to the risk of a preterm birth [26].

An umbrella review of 1480 studies recently identified 166 risk factors for preterm birth [27]. Seven factors with robust evidence include: isolated single umbilical artery, amphetamine exposure, low gestational weight gain, maternal personality disorder, sleep-disordered breathing, vacuum aspiration for pregnancy termination, and an interpregnancy interval of less than 6 months after miscarriage. The associations between preterm birth and various maternal, pregnancy, and environmental factors suggest a multifactorial cause, involving pathophysiological changes in both mother and fetus.

### 2.2. Normal Course of Kidney Development

Kidney development in humans begins around weeks 3–4 of gestation and continues until approximately 36 weeks [28]. This process involves the formation of three kidney structures from the posterior intermediate mesoderm: the pronephros, mesonephros, and metanephros. The pronephros and mesonephros are primitive structures that regress, while the metanephros evolves into the definitive kidneys.

Metanephric kidney development starts with the formation and elongation of the ureteric bud (UB), which invades the adjacent metanephric mesenchyme (MM) [29]. The MM differentiates into nephrons, while the UB branches to form the collecting ducts. Renal vesicles, formed through mesenchyme-to-epithelium conversion, are precursors to the nephrons. Branching morphogenesis of the UB establishes the collecting duct system, which ultimately supports nephron formation.

A nephron is the kidney’s basic functional unit, and nephron endowment refers to the total number of nephrons present at birth, reflecting the success of nephrogenesis. Human kidneys typically contain about 1 million nephrons, though this number varies significantly among individuals [14]. Nephron development increases exponentially between 18 and 32 weeks of gestation, with nephrogenesis generally concluding by term birth [30]. After birth, the kidney continues to grow [31]. The glomerular filtration rate (GFR) doubles in the first 2 weeks of life, from 20 mL/min/1.73 m^2^ at birth, and reaches adult levels by age two [32].

Nephrogenesis in rodents, while similar to humans, occurs at a faster pace. After birth, kidney development continues with the maturation of nephrons, which extends into the first 1–2 weeks postnatally. By embryonic day 16, both afferent and efferent nerves are present within the developing kidney [33]. These nerves begin to infiltrate the kidney during late gestation, reach the outer cortical renal arterioles by the first 1–2 weeks after birth, and continue their maturation throughout postnatal development [34]. Thus, suboptimal conditions during both gestation and the early postnatal period can significantly affect kidney development in rodents.

### 2.3. Preterm Birth, Low Nephron Numbers, and CAKUT

Most nephron formation occurs during the third trimester of pregnancy, a critical period when preterm infants are often born. Adverse events occurring before the completion of nephrogenesis can compromise kidney development, resulting in reduced nephron numbers and a range of clinical phenotypes, namely congenital anomalies of the kidney and urinary tract (CAKUT) [35,36]. A deficit in nephron numbers can lead to increased glomerular capillary pressure and hyperfiltration, which in turn causes compensatory glomerular and tubular hypertrophy. This creates a vicious cycle of further nephron loss over time [37].

CKD often results from a series of insults or “hits” [16]. In this context, a programmed low nephron endowment can be considered an initial “first hit” to the kidney. This reduced nephron number leaves the remaining glomeruli more vulnerable to environmental stressors and renal injuries. Consequently, the kidneys are at a heightened risk of developing CKD when exposed to additional insults later in life.

Preterm infants are particularly at risk for low nephron endowment due to factors such as intrauterine growth retardation (IUGR), compromised pregnancy, inadequate postnatal nutrition, and the use of drugs like NSAIDs after birth [13,14,15,37,38]. Prematurity and LBW are strong clinical indicators of low nephron endowment [30]. Both conditions are also significant risk factors for CAKUT, a major cause of CKD in children [36]. Given that CAKUT encompasses a broad spectrum of renal structural malformations and varying nephron deficits, the low nephron endowment associated with CAKUT may contribute to the high prevalence of CKD among individuals with these anomalies.

Autopsy studies of preterm human kidneys have revealed a reduced number of mature nephrons [39]. Nevertheless, directly determining nephron numbers in vivo remains a challenge. Although ferritin-based nanoparticles have shown some promise as targeted magnetic resonance imaging (MRI) contrast agents for estimating nephron numbers in human kidneys [40], validating non-invasive methods for assessing nephron endowment in vivo requires further research. Currently, nephron endowment is primarily estimated through surrogate markers, such as reduced kidney mass and volume. Clinical indicators of a diminished renal reserve in newborns and children include low renal volume, as assessed by ultrasound measurements of renal length [41], and three-dimensional ultrasound volume [42]. A kidney biopsy, while accurate, should be reserved for selected cases due to ethical concerns, and is not suitable for widespread application.

Despite having fewer nephrons, preterm infants often achieve a GFR comparable to that of term neonates through compensatory mechanisms, such as single nephron hyperfiltration. However, this adaptation can lead to long-term consequences, including glomerular damage, proteinuria, and hypertension. Over time, these conditions increase the risk of developing CKD.

## 3. Preterm Birth and Later-Life CKD: Evidence from Human Studies

Human research has consistently demonstrated a range of adverse kidney outcomes in individuals born prematurely, affecting them from infancy through to older adulthood (see Table 1). These outcomes include reduced kidney volume, diminished kidney function as indicated by an eGFR, elevated levels of creatinine (Cr) or cystatin C, increased blood pressure (BP), microalbuminuria, and a heightened risk of developing CKD.

Horie et al. retrospectively analyzed the eGFR in 168 neonates born prematurely. They found that the eGFR at 2 years of age was significantly correlated with gestational age [43]. Kwinta and colleagues investigated a regional cohort of 78 infants born at a median gestational age of 27 weeks with an extremely low birth weight (ELBW). They found that, by school age, cystatin C levels and kidney volume were significantly lower in these ELBW children. However, BP and microalbuminuria were not significantly different [44].

Several studies have evaluated long-term kidney outcomes in adolescents born preterm. One study found that infants born at extremely low gestational ages (<28 weeks) exhibited a lower eGFR, reduced kidney volume, and elevated cystatin C levels by the age of 11 years [45,46]. Similarly, another study involving 93 adolescents born at gestational ages less than 34 weeks reported that the preterm birth was associated with a decreased eGFR, shorter kidney length, and elevated systolic and diastolic BPs by the same age [47]. Interestingly, the higher BP was linked to lower plasma renin activity, indicating that hypertension after preterm birth is likely not mediated by the renin–angiotensin system (RAS), contrary to previous proposals.

Rodríguez-Soriano et al. followed 40 infants born at less than 35 weeks’ gestational age up to ages 6 to 12 years, finding that these infants had a lower eGFR compared to controls [48]. Similarly, South and colleagues observed that, at age 14, prematurely born children had both a lower eGFR and higher BP compared to healthy term-born controls [49]. Sanderson et al. reported that, among 42 premature participants, 14% had low kidney volume, 11.9% had microalbuminuria, and 33.3% had an elevated BP at age 15 [50]. Additionally, a large prospective study of 422 infants born at less than 32 weeks’ gestational age found that birth weight was positively associated with an eGFR and negatively associated with microalbuminuria [51].

Two significant studies underscore the long-term impact of preterm birth on kidney health in adults. A large Swedish cohort study, encompassing over 4 million live births, revealed that young adults (up to age 43) born preterm faced a 2- to 3-fold higher risk of CKD compared with those born at term, with CKD risk inversely related to gestational age [52]. Specifically, at ages 18–29, the adjusted hazard ratios (HR) for CKD were 1.28 for preterm and 2.45 for extremely preterm births, compared to term births. At ages 30–43, the hazard ratios were 1.25 and 1.68 for preterm and extremely preterm births, respectively [52].

Likewise, the Helsinki Birth Cohort Study, which followed 20,431 participants from birth until death or age 86, revealed a significant association between CKD and being born before 34 weeks of gestation, with a median age of CKD onset at 65 years. The hazard ratio for this association was 2.6 (95% CI = 1.3–4.6), and it was notably stronger in female infants, showing a hazard ratio of 3.2 (95% CI = 1.4–7.4) [53].

In a cohort study of 2,679,967 individuals aged 18–50, with 1181 developing end-stage kidney disease (ESKD), it was found that having at least two risk factors—LBW, small for gestational age (SGA), or preterm birth—was linked to an increased risk of ESKD. However, preterm birth alone was not a significant risk factor for ESKD [54]. This highlights the need to distinguish between different risk factors rather than treating prematurity as a single category.

These epidemiological findings indicate that, in addition to a heightened risk for CKD, preterm birth is associated with reduced kidney volume, hypertension, and microalbuminuria, all of which are major risk factors for developing CKD.

## 4. Preterm Birth and Kidney Programming: Evidence from Animal Studies

Despite the assessment of preterm birth’s impact on kidney disease in numerous human studies, the specific interventions needed to establish causation and elucidate the underlying pathophysiological mechanisms are not yet fully understood. Consequently, our current knowledge about the types of insults driving kidney programming due to prematurity, the critical windows of vulnerability, and potential mechanisms largely comes from animal model studies.

### 4.1. Animal Models of Preterm Birth

Recent research aimed at understanding preterm birth has increasingly utilized animal models to replicate its pathophysiology [55,56,57]. While many studies have focused on infection- and inflammation-based models—using bacteria or their products, as well as individual cytokines—these approaches address only a subset of the various triggers for preterm birth [56,57]. Moreover, the variability in outcomes and maternal morbidity across different laboratories highlights inconsistencies with human preterm birth.

Dysregulation of maternal immune responses has been implicated in preterm birth, with early immune disturbances induced by factors such as maternal stress, infection, diet, and environmental pollutants potentially leading to impaired immune tolerance and excessive inflammation [57]. Thus, there is a pressing need for animal models that more accurately reflect the diverse factors contributing to human preterm birth [55].

Given the close relationship between preterm birth and intrauterine growth restriction (IUGR), prenatal interventions used in animal models of IUGR may offer valuable insights into preterm birth. A systematic review highlighted that many studies involving IUGR also resulted in preterm delivery, with the median gestational age at delivery being around 95% of the full pregnancy length [58].

In rat models, the most common methods for inducing IUGR were surgical (23%), followed by pharmacological (22%), nutritional (19%), and toxic (19%) approaches [58]. Various risk factors associated with human preterm birth have been used to induce IUGR in animal models, including maternal hypoxia [59], dexamethasone administration [60], maternal diabetes [61], maternal smoking [62], pre-eclampsia [63], maternal malnutrition [64], placental insufficiency [65], and maternal inflammation [66]. Despite these advances, most studies primarily report on delivery or immediate neonatal outcomes, with long-term adverse effects remaining underexplored [58].

### 4.2. Animal Models of Kidney Programming

Several animal studies have established a link between early-life insults, kidney programming, and the development of kidney disease in later life. These studies often focus on kidney programming associated with low nephron numbers, as summarized in Table 2 [67,68,69,70,71,72,73,74,75,76,77,78,79,80,81].

### 4.3. Low Nephron Numbers

Unlike humans, kidney development in rodents continues into the first 1–2 postnatal weeks, making the period of pregnancy and early lactation critical for nephrogenesis. Adverse conditions during this time can impair nephron formation, leading to permanent kidney programming and increased susceptibility to adulthood kidney disease. Table 2 outlines various environmental factors examined in relation to kidney programming and reduced nephron endowment. These factors include maternal nutrition [67,68,69,70,71,72], maternal illness and obstetric complications [73,74,75], environmental chemical exposure [76,77], and medication use [78,79,80,81].

Reduced nephron numbers, a condition that can develop from birth through adulthood, is seen in various experimental models of kidney programming. Notably, even brief exposure to adverse conditions during the nephrogenesis period—sometimes as short as a few days—can result in permanently low nephron endowment [75,76,79]. For example, in rats, administering dexamethasone for just 2 days during embryonic days 13–14 or 17–18 leads to a reduced nephron number in adult offspring [79]. These findings highlight a critical window of vulnerability for kidney development.

The major adverse outcomes associated with kidney programming and low nephron endowment include glomerular hypertrophy [61,69,71,72,73,74,78,81] and elevated BP [67,68,69,70,71,73,77,80], with subsequent reductions in the GFR [74,75,76,77,78] and tubulointerstitial injury [69,73]. While a decline in nephron numbers, if not accompanied by compensatory hypertrophy, is expected to lead to a reduction in the GFR. Variations in the GFR observed across different kidney programming models reveal that it can be reduced [74,75,76,77,78], unchanged [69,70,73,79,81], or even increased [72].

These discrepancies suggest that the degree of compensatory hypertrophy in response to low nephron endowment varies among different models and stages of life. Therefore, low nephron endowment alone does not account for all programmed processes related to kidney disease development. This indicates that kidney programming involves factors beyond just nephron number, and further investigation into additional mechanisms is needed.

### 4.4. Prenatal Hits: Types of Maternal Insults

Table 1 demonstrates that nutritional imbalance is the most prevalent factor inducing kidney programming. Nutritional insults can be categorized into various models based on their manipulation of specific dietary elements: sodium intake [67], protein intake [68,70], calorie intake [69], iron intake [71], and multiple nutrient intake [72]. Given the crucial role of nutrients in fetal development, previous studies have used both excessive and insufficient levels of specific nutrients to create animal models for investigating kidney programming [82]. Since maternal nutritional imbalances not only contribute to preterm birth but to kidney programming as well, maternal nutritional interventions could potentially serve as a strategy to prevent both conditions and reduce the risk of subsequent CKD.

In the context of maternal illness and obstetrical complications, such as diabetes [73], reduced uterine perfusion [74] and inflammation [75] have been reported to impair nephrogenesis and cause low nephron endorsement (Table 1). Notably, pre-eclampsia [83] maternal hypoxia [84], and maternal CKD [85] have also been established to induce kidney programming.

In a rat model of maternal ethanol exposure, a decreased nephron number and diminished renal function were observed in adult offspring, potentially due to impaired ureteric branching morphogenesis [76]. Similarly, a model involving maternal exposure to di-2-ethylhexylphthalate (DEHP) revealed that adult offspring exhibited reduced kidney function and hypertension, accompanied by dysregulation of several genes involved in nephrogenesis [77]. These findings indicate that certain chemicals can disrupt nephrogenesis during kidney development, leading to a lower nephron endowment and CAKUT [86,87]. Additionally, other environmental chemicals, including bisphenol A (BPA) [88], 2,3,7,8-tetrachlorodibenzo-p-dioxin (TCDD) [89], di-n-butyl phthalate [90], and smoking [91], have also been implicated in kidney programming.

Notably, BPA, TCDD, and phthalates are recognized as endocrine-disrupting chemicals (EDCs). These substances interfere with hormone signaling pathways, adversely impacting kidney development and function [87]. In addition to contributing to a reduced nephron number, the effects of endocrine disruption can extend to subsequent generations. While emerging evidence supports the association between EDC exposure during fetal life and an increased risk of CKD later in life, further research is essential to clarify the extent to which these EDCs affect kidney structure and function across generations.

The existing literature suggests that many medications administrated to pregnant women may affect kidney development and cause CAKUT [92]. These medications cover cyclosporine, aminoglycosides, ACE inhibitor (ACEI)/angiotensin receptor blockers (ARBs), NSAIDs, dexamethasone, furosemide, Adriamycin, anti-epileptic drugs, and cyclophosphamide [92].

Table 1 highlights that both glucocorticoids and cyclosporine can reduce nephron numbers [80,81]. A recent study involving 23,363 singleton-born children found that gestational exposure to systemic glucocorticoids significantly increased the risk of childhood CKD. This risk was particularly pronounced in cases of preterm birth, exposure during the second trimester, and when the total dose exceeded 24 mg of a hydrocortisone equivalent [93]. Animal studies corroborated these findings, showing that administering dexamethasone for 2 days during the embryonic days 13–14 or 17–18, or for 1 week before delivery, resulted in reduced nephron numbers in adult rat offspring [78,79,80]. Although the nephron number was not assessed, dexamethasone administration on postnatal days 1–3 also led to kidney programming [94]. Thus, glucocorticoid exposure during nephrogenesis, whether prenatal or postnatal, adversely affects kidney outcomes in adult offspring.

Similarly, the use of cyclosporine during gestation has been linked to reduced nephron numbers in animal models [81] and linked to poor neonatal outcomes such as preterm birth and LBW [95]. Furthermore, the use of ACEI and ARBs is avoided in pregnant women because of their association with ACEI/ARB fetopathy and renal maldevelopment [96]. The suppression of the intrarenal RAS by these medications is known to disrupt normal kidney development [12].

## 5. Hypothetical Mechanisms of Kidney Programming

Several hypothetical mechanisms have been proposed to explain kidney programming, including oxidative stress, aberrant RAS, glucocorticoid effects, epigenetic regulation, gut microbiota dysbiosis, and sex differences [8,9,10,12,13,97]. Preterm birth is particularly associated with these mechanisms.

### 5.1. Oxidative Stress

A balance between oxidants and antioxidants is crucial for proper fetal development [98]. An imbalance in this system leads to oxidative stress, which can particularly affect the developing kidney, making it susceptible to oxidative injury. Several animal models, as detailed in Table 2, demonstrate that oxidative stress-related kidney programming can result from conditions such as maternal diabetes [73], pre-eclampsia [83], maternal high-fructose diet [99], and prenatal dexamethasone exposure [100]. Additionally, a range of environmental factors—including imbalanced maternal nutrition, maternal illness, exposure to environmental chemicals, and medication use—can contribute to oxidative-stress-related renal programming [101]. These factors are often associated with preterm birth. By contrast, antioxidant therapies have shown promise in mitigating kidney programming and preventing kidney disease [101].

### 5.2. Aberrant RAS

The RAS is essential for nephrogenesis and renal function [102,103]. It includes the classical RAS, which involves the angiotensin converting enzyme (ACE)-angiotensin (Ang) II-angiotensin type 1 receptor (AT1R) axis promoting vasoconstriction, and the non-classical RAS, with the ACE2-Ang-(1-7)-Mas receptor leading to vasodilation [103]. Research on these RAS axes in fetal programming has shown mixed results, with conflicting reports on the regulation of the RAS components [12]. Generally, RAS expression decreases at birth, but may become inappropriately high in adulthood, potentially affecting kidney development [12].

In models of prenatal LPS exposure, alterations in the intrarenal RAS were linked to kidney dysfunction in adult rat offspring [75]. Similarly, in maternal DEHP exposure models, an impaired kidney development and subsequent kidney disease have been associated with RAS inhibition [77]. The early-life blockade of the classical RAS between 2 and 4 weeks has shown potential in mitigating kidney programming and enhancing kidney health [104,105,106,107]. Notably, an aberrant RAS may contribute to preterm birth by inducing inflammation and disrupting fetal membrane integrity during pregnancy [108]. While targeting the RAS to prevent adverse kidney outcomes related to kidney programming has shown promise [109], its potential for preventing preterm birth has yet to be explored.

### 5.3. Glucocorticoid Programming

Glucocorticoids are crucial for normal fetal development and organogenesis [110,111,112]. Typically, fetal glucocorticoid levels are much lower than maternal levels at term, due to the placental enzyme 11β-hydroxysteroid dehydrogenase type 2 (11β-HSD2), which inactivates active glucocorticoids [112]. Adverse conditions such as maternal malnutrition, pre-eclampsia, and maternal stress can inhibit 11β-HSD2 [112], leading to excessive fetal glucocorticoid exposure. Early-life glucocorticoid exposure has been shown to reduce nephron numbers, induce kidney programming, and increase the risk of childhood CKD [78,79,80,93,94]. Additionally, RNA sequencing studies have revealed that such exposure significantly alters renal transcripts in offspring, with 431 renal transcripts affected at 16 weeks, including genes involved in branching morphogenesis [113].

### 5.4. Epigenetic Regulation

Epigenetic regulation has a significant role in fetal programming [114] and preterm birth [115]. Epigenetics involves modifications in gene expression that do not alter the DNA sequence and can be influenced by environmental factors [116]. Major epigenetic mechanisms contain histone modification, DNA methylation, and microRNA (miRNA)-mediated silencing [116].

In our previous work using NGS to analyze RNA transcript levels in two-week-old offspring kidneys exposed to maternal insults, we identified 809, 965, 356, and 272 differentially expressed genes (DEGs) in the caloric restriction, diabetes, high-fructose, and high salt models, respectively [117,118]. Research has shown that the interplay between the RAS and histone deacetylases (HDACs) influences ureteric bud branching during nephrogenesis [119]. HDACs have been implicated in regulating several RAS genes, including angiotensin (*Agt*), renin, ACE, and AT1R [119]. Additionally, HDAC inhibitors have been found to prevent neonatal dexamethasone-induced hypertension by reducing levels of *Agt, Ace,* and *Ace2* [120]. Further studies revealed that genes associated with histone modification, such as *Brwd1*, *Dnmt3l*, *Chd2*, *Brdt*, *Hdac9*, *Myst2*, and *Hdac11*, are regulated in offspring kidneys from rats fed a high-fructose diet [121].

Given that certain dietary components may induce protective epigenetic modifications throughout life [122] and given the potential therapeutic use of HDAC inhibitors for preterm birth prevention [123], exploring epigenetic targeting to prevent preterm birth and kidney programming is a promising research area.

### 5.5. Gut Microbiota Dysbiosis

Recent research has increasingly focused on the role of gut microbiota in kidney programming and the development of CKD later in life [97,124,125]. Gut microbiota and their metabolites can influence the function of different organs, including the kidneys, via the bloodstream. The gut-kidney axis in CKD involves mechanisms such as gut barrier dysfunction, inflammation, immune response, altered microbiota compositions, dysregulated short-chain fatty acids (SCFAs) and their receptors, trimethylamine-N-oxide, and uremic toxins [124]. Conversely, gut microbiota-based therapies have been explored for early CKD prevention in animal models [97].

Maternal factors and early-life events shape the infant gut microbiome [126]. Preterm infants, exposed to unique environmental conditions, have distinct microbiota compositions, though the impact on long-term health remains unclear. Notably, probiotics have shown benefits for the preterm gut microbiome and immune function [127]. Given that such early interventions targeting the gut microbiota, including prebiotics, probiotics, and postbiotics, have demonstrated potential in mitigating kidney programming-related adverse outcomes in animal models [97], further research is needed to determine if manipulating the microbiota of preterm infants can prevent CKD in adulthood.

However, comprehensive animal studies that simultaneously address these mechanisms to explore their impact on kidney programming and preterm birth are still lacking. Investigating a broad range of mechanisms and evaluating preventive therapies in animal models of preterm birth to prevent CKD later in life remains an ambitious and distant goal.

## 6. Postnatal Hits: What Preterm Infants May Face?

Kidney programming and a low nephron number amplify the impact and consequences of postnatal renal insults. The endogenous and iatrogenic factors in the postnatal care environment that contribute to a second hit for CKD later in life are acute kidney injury (AKI), nephrotoxic drugs, preterm nutrition, and catch-up growth.

### 6.1. Acute Kidney Injury

AKI, often involving neonates admitted to the neonatal intensive care unit (NICU), is related to poor outcomes in premature neonates [128]. A systemic review including 50 studies of 10,744 patients revealed that the overall rate of AKI from the pooled results was 25% [129]. Additionally, AKI was associated with a high mortality rate among preterm neonates. Major risk factors for AKI in preterm neonates include the following: a LBW, sepsis, a low Apgar score, mechanical ventilation, patent ductus arteriosus (PDA), vasoactive drugs, NSAID treatment, and nephrotoxic antibiotics [129].

Premature kidneys in LBW neonates are especially vulnerable to injury, particularly when exposed to additional stressors. Premature neonates are at a heightened risk for sepsis, which can trigger a systemic inflammatory response, and is associated with a high mortality risk of around 70% in the NICU [130]. The inflammatory cytokines released during sepsis, combined with potential kidney hypo-perfusion, can exacerbate renal injury through oxidative stress and cellular apoptosis [131].

Neonates with low Apgar scores are also at risk due to acidosis and hypoxia [132,133]. Mechanical ventilation introduces further risks, including hemodynamic instability and ventilator-induced lung injury, which can lead to systemic inflammation affecting renal function [133]. PDA can cause relative hypovolemia and hypoperfusion, leading to increased production of vasodilatory prostaglandins. While NSAIDs may help manage PDA-related vasodilation, they can also impair renal perfusion, potentially causing additional kidney damage [132]. Furthermore, antibiotics used in sepsis treatment may have nephrotoxic effects, worsening kidney damage already caused by hypotension, hypoperfusion, and inflammation.

In addition to PDA, various prematurity-related complications impact multiple organ systems, including necrotizing enterocolitis (NEC), bronchopulmonary dysplasia, intraventricular hemorrhage, hypoxic-ischemic encephalopathy (HIE), and retinopathy of prematurity [134]. These conditions can reduce blood flow and cause hypotension, which may impair kidney function and lead to AKI. For instance, NEC induces systemic inflammation that can further exacerbate AKI.

The distinction between prematurity-related complications and AKI in preterm infants is often blurred, as many prematurity-associated conditions can directly contribute to or worsen AKI. The immaturity of the kidneys in these infants makes them particularly vulnerable to these complications [135].

Furthermore, management strategies for prematurity-related issues can sometimes have adverse effects on renal function, compounding the risk of AKI. The interaction of these risk factors often results in a cumulative effect, significantly increasing the likelihood of AKI in this sensitive population. Recurrent AKI is not uncommon among preterm neonates and can lead to poorer outcomes in the NICU, including an increased risk of progression from acute damage to CKD [136].

### 6.2. Nephrotoxic Drugs

Nephrotoxic drugs increase the risk of AKI [137]. Critically ill neonates face an increased risk of developing AKI and are often prescribed medications that further raise this risk. Notably, the highest exposure levels were observed in the smallest and most immature neonates, particularly those who developed AKI. Nephrotoxic drugs commonly used in NICU include antibiotics, antifungals, NSAID drugs, and diuretics [138,139].

Nephrotoxicity is influenced by several factors, including age, existing comorbidities, drug dosage, and any concurrent medications. Notably, over 80% of neonates receive one or more potentially nephrotoxic drugs [140,141]. A lower gestational age results in a reduced clearance of aminoglycosides, leading to potentially toxic drug levels [142]. The same issue applies to vancomycin. Among antibiotics, the risk of nephrotoxicity varies: carbapenems pose the highest risk, followed by cephalosporins, penicillin, and monobactams. Additionally, VLBW infants are susceptible to systemic fungal infections. Amphotericin B, a potent antifungal used for treating invasive candidiasis in neonates, is less nephrotoxic in its liposomal form (AmBisome) [143].

As stated earlier, NSAIDs like ibuprofen and indomethacin are commonly used to manage PDA. Both drugs affect kidney function and renal drug clearance. Ibuprofen has been linked to a 21% reduction in amikacin clearance and an 18% reduction in vancomycin clearance, while indomethacin has a more pronounced effect, significantly reducing vancomycin clearance [144].

In neonates with oliguric AKI, loop diuretics, such as furosemide, are commonly used to enhance diuresis and prevent fluid overload [145]. Despite their frequent off-label use in the NICU for various conditions, studies have not consistently shown that diuretics improve key clinical outcomes [146]. While diuretics can influence renal function, particularly in preterm neonates, their nephrotoxic effects vary depending on the specific drug, dosage, and the infant’s overall health [146].

Reducing exposure to nephrotoxic drugs has been shown to decrease AKI rates in the NICU [147]. Therefore, implementing preventive strategies to minimize nephrotoxic drug exposure is crucial for reducing the risk of AKI and potentially preventing CKD later in life.

### 6.3. Preterm Nutrition and Catch-Up Growth

Infants born preterm have heightened nutritional needs compared to term infants of the same postnatal age, making them more susceptible to nutrient deficiencies and challenges in maintaining adequate nutrition intake. Therefore, ensuring optimal nutrition is critical for their growth and development.

Several hypotheses within the DOHaD research, including the thrifty phenotype, predictive adaptive responses, and the catch-up growth hypothesis, seek to explain the link between early life malnutrition and later chronic diseases [148,149]. Thrift theory emphasizes the complex relationship between early nutritional experiences and later health, suggesting that adaptive mechanisms that once promoted survival can increase susceptibility to CKD in today’s context [148]. Additionally, rapid weight gain—often referred to as catch-up growth—has been associated with higher risks of chronic diseases in adulthood [149]. This connection further illustrates how early life adversities can predispose individuals to CKD later in life.

Despite various nutrition guidelines for preterm infants, there remains a lack of consensus on the best practices, resulting in significant variability in clinical settings [150]. A recent systematic review of 27 guidelines revealed inconsistencies in the certainty of evidence supporting different recommendations, highlighting gaps with very low certainty [151].

The growth trajectory during the first two years of life is pivotal for a child’s future development and risk of chronic diseases [152]. Preterm infants, particularly those with accelerated catch-up growth, face a higher risk of metabolic syndrome, which includes insulin resistance, central adiposity, and elevated BP from early childhood [153,154]. These early-life growth patterns play a crucial role in determining cardiometabolic health throughout life [155]. Consequently, monitoring the growth trajectory of preterm infants during their first two years is essential. Although direct links between early growth patterns in preterm infants and CKD in adulthood are not extensively studied, evidence suggests that BMI trajectories from childhood to midlife may be connected to subclinical kidney damage later in life [156]. Recent research emphasizes the interconnectedness of cardiovascular, kidney, and metabolic conditions, leading to the concept of cardiovascular–kidney–metabolic syndrome [157]. While the precise mechanisms and impact of cardiovascular and metabolic disorders on CKD are still under investigation, early interventions targeting these interrelated conditions may offer opportunities to mitigate their long-term adverse effects [158].

## 7. Recommended Actions: What Should We Do?

**Figure 1:**
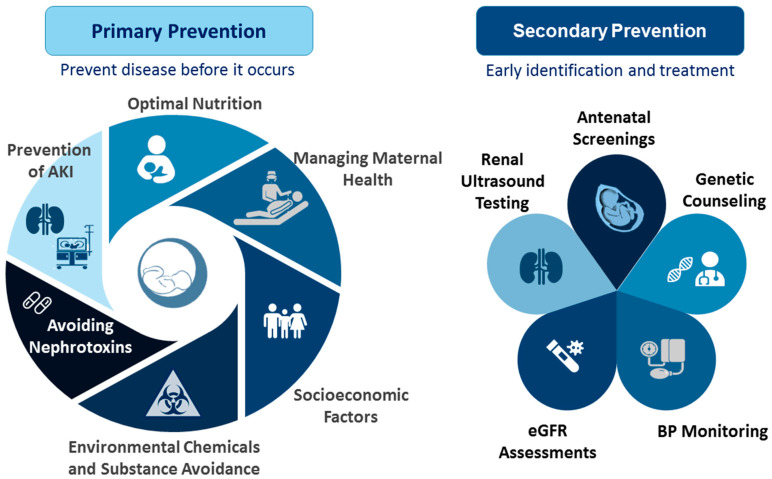
Primary and secondary prevention to improve kidney health in preterm infants: outline of key recommended actions.

In 2020, World Kidney Day highlighted the significance of preventive strategies—primary, secondary, or tertiary—for enhancing kidney health [159]. Due to the complex, multifaceted nature of CKD, a comprehensive approach is essential. While tertiary prevention targets advanced CKD and its associated comorbidities, primary and secondary prevention are particularly crucial for preterm infants during the critical first 1000 days of life. The key recommended actions are illustrated in Figure 1.

Primary prevention focuses on preventing kidney disease by targeting modifiable risk factors. These factors, which simultaneously affect kidney programming and the risk of preterm birth, include maternal malnutrition, illness, pregnancy complications, exposure to environmental chemicals, and medication use. Key strategies for primary prevention include the following: (1) Optimal Nutrition: Ensuring adequate nutrition from pregnancy through infancy is crucial. For preterm infants, this includes optimizing breastfeeding practices and monitoring growth to support linear development while avoiding excessive catch-up growth [160,161]. The WHO and UNICEF advocate for exclusive breastfeeding during the first six months of life, followed by continued breastfeeding for up to two years or longer [162]. (2) Managing Maternal Health: Women should undergo screening for known risk factors for preterm birth either before conception or early in gestation. In women at high risk of preterm birth, preventive interventions should be applied if possible [163]. Controlling maternal illnesses and managing pregnancy complications are essential for normal delivery and fetal development. Additionally, protecting preterm infants from infections through timely vaccinations is vital [164]. (3) Socioeconomic Factors: Enhancing access to family planning, education, and poverty reduction can significantly impact both maternal and fetal health [165]. (4) Environmental Chemicals and Substance Avoidance: Preventing exposure to environmental chemicals and avoiding substance abuse are crucial for reducing the risks associated with preterm birth and enhancing kidney health. (5) Avoiding Nephrotoxins: Nephrotoxic drugs should be avoided whenever possible and used only as a last resort when no alternatives are available. It is important to avoid nephrotoxic drugs during pregnancy and in NICUs to prevent AKI. Finally, (6) Prevention of AKI: Every effort should be made to prevent AKI, including optimizing fluid management, minimizing the use of nephrotoxic medications, monitoring aminoglycoside levels during extended treatments, adjusting drug dosages and intervals in cases of established nephrotoxicity, enhancing nutritional support, and conducting renal ultrasounds to evaluate CAKUT for timely intervention [166,167]. By addressing these factors, we can better support kidney health in preterm infants and reduce the risk of kidney disease from early life [168].

Secondary prevention focuses on the early detection and treatment of kidney disease. Early identification of CKD can have substantial public health benefits; however, many countries lack sufficient CKD surveillance systems [169]. For preterm infants, crucial screening services include antenatal screenings, renal ultrasound testing, eGFR assessments, BP monitoring, and genetic counseling. While various potential biomarkers for AKI have been investigated, none have yet been validated sufficiently, particularly for preterm neonates [170,171]. Additionally, there is a need for a reliable, noninvasive tool to measure nephron numbers accurately for both clinical and research purposes [172].

Animal studies have yielded crucial insights into reprogramming strategies for preventing kidney disease. Oxidative stress, a key factor in kidney programming, has been targeted using antioxidants in various animal models [173]. Notable antioxidants studied include L-taurine [174], folate [175], N-acetylcysteine [176], melatonin [177], resveratrol [178], vitamin E [179], polyphenol [180], and selenium [181]. Emerging evidence indicates that antioxidant therapies could be beneficial for preventing preterm birth and managing prematurity-associated conditions and their long-term effects [182,183]. These findings suggest that addressing oxidative stress through antioxidant interventions in early life may help prevent both preterm birth and CKD later in life.

Moreover, common mechanisms underlying preterm birth and kidney programming provide additional targets for improving kidney health in preterm infants. Despite significant progress in animal research, identifying research gaps and achieving meaningful clinical translation remain essential priorities.

## 8. Strengths and Limitations

This review highlights several significant strengths. Firstly, it effectively identifies key knowledge gaps regarding the renal consequences of preterm birth, emphasizing the complexity of kidney development and the various influencing factors while proposing actionable research directions. Secondly, the focus on developing non-invasive methods for assessing the nephron count and function suggests promising avenues for enhancing clinical care and monitoring at-risk populations. Thirdly, the review underscores the importance of the early identification of specific risk factors and advocates for longitudinal studies promoting tailored research approaches to mitigate adverse kidney outcomes in preterm infants. Lastly, it outlines crucial recommended actions that can aid in the primary and secondary prevention of kidney health issues in this vulnerable population.

However, there are notable limitations. Significant knowledge gaps remain regarding the molecular mechanisms of kidney development and the effects of preterm birth, which may impede progress in the field. Additionally, variability in the doses and durations of maternal insults across different animal models complicates the establishment of universal conclusions and may obscure specific risk factors. There is also a pressing need for more longitudinal studies to draw definitive conclusions about the long-term renal outcomes for preterm infants. Furthermore, the interplay of genetic, epigenetic, and environmental factors adds layers of complexity to the research, making it difficult to isolate specific influences on nephron endowment and kidney health. Current evidence on sex-specific and population-specific effects related to preterm birth and adult kidney disease remains unclear, highlighting the necessity for more nuanced research designs.

## 9. Conclusions and Perspectives

**Figure 2:**
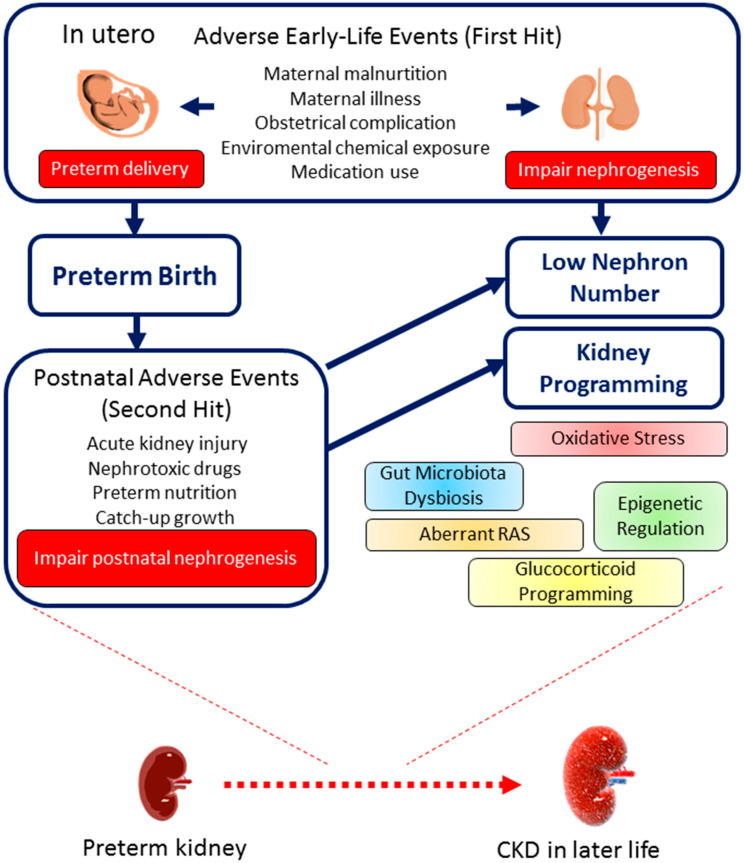
The consequences of preterm birth on kidney health and disease throughout the life course. An outline of the early-life (first hit) and postnatal (second hit) events that contribute to low nephron numbers, kidney programming, and the development of chronic kidney disease (CKD) later in life.

The entire framework surrounding early-life and postnatal events influencing preterm kidney development and their long-term consequences is depicted in Figure 2. Kidney programming illustrates how prenatal stressors serve as an initial “hit”, resulting in reduced nephron formation and triggering pathological mechanisms. Subsequent exposure to adverse postnatal factors, acting as a second “hit”, can lead to lasting clinical complications in the kidneys of preterm children.

Neonatologists are typically attentive to the immediate risks of prematurity in NICU care, but the long-term risk of CKD often receives less attention. This risk could be substantial and may develop over time by kidney programming, especially given that many premature infants who have survived neonatal care are now reaching adulthood. We might already be encountering a concealed epidemic of CKD within this cohort.

The reviewed evidence underscores the significant risk of CKD in preterm infants and emphasizes the need to understand the impacts of maternal and postnatal factors, kidney programming, and reduced nephron numbers. Despite the promising findings in animal studies, high-quality, long-term research and guidelines to inform pediatricians on managing these risks effectively are lacking. Tackling these unmet needs could lead to the identification of cost-effective strategies and optimized interventions to reduce or prevent the developmental programming of CKD later in life, especially for children born preterm. We urge professional organizations, policymakers, neonatologists, pediatricians, and funding agencies to prioritize this critical issue and ensure that advancements in kidney health keep pace with improvements in the care of premature infants.

## Figures and Tables

**Table 1:** Human studies linking preterm birth to adverse kidney outcomes.

Study	Country	Study Population	Sample Size	Age at Measure (Years)	Adverse Kidney Outcomes	Ref.
Horie et al.	Japan	GA < 35 weeks	168	2	Low eGFR	[43]
Kwinta et al.	Poland	Median GA 27 weeks	78	6–7	Low kidney volume and high cystatin C	[44]
Vollsæter et al.	Norway	GA < 28 weeks	57	11	Low eGFR	[45]
Starzec et al.	Poland	GA < 28 weeks	64	11	Low kidney volume and high cystatin C	[46]
Raaijmakers et al.	Belgium	GA < 34 weeks	93	11	Low eGFR and kidney length, and high systolic and diastolic BP	[47]
Rodríguez-Soriano et al.	Spain	GA < 35 weeks	40	6–12	Low eGFR	[48]
South et al.	USA	GA < 37 weeks	96	14	Low eGFR and high systolic and diastolic BP	[49]
Sanderson et al.	USA	GA < 28 weeks	42	15	Low kidney volume, microalbuminuria, and elevated BP	[50]
Keijzer-Veen et al.	Netherlands	GA < 32 weeks	442	19	Low eGFR and microalbuminuria	[51]
Crump et al.	Sweden	GA < 28 weeks	4,186,615	43	CKD	[52]
Eriksson et al.	Finland	GA < 34 weeks	20,431	86 or death	CKD	[53]

**Table 2:** Animal models of kidney programming with low nephron numbers.

Experimental Model	Reduced Nephron Number	Age at Evaluation (Weeks)	Kidney Outcomes	Ref.
Maternal nutrition				
Low sodium diet (0.07%) during gestation and breastfeeding	Yes	1	↑ BP at 5 mo	[67]
High sodium diet (3%) during gestation and breastfeeding	Yes	1	Glomerular hypertrophy, ↑ BP at 5 mo	[67]
Low protein diet (8% protein) during lactation	Yes	8	↑ BP at 5 mo	[68]
50% caloric restriction during gestation and breastfeeding	Yes	12	↔ GFR, glomerular hypertrophy, ↑ BP, tubulointerstitial injury	[69]
Low protein diet (8.5% protein) during gestation	Yes	22	↔ GFR, ↑ BP	[70]
Iron restriction diet (3 mg/kg diet) from 1 wk before mating and through pregnancy	Yes	72	Glomerular hypertrophy, ↑ BP	[71]
Multi-deficient diet during gestation	Yes	12	↑ GFR, glomerular hypertrophy	[72]
Maternal illness and obstetrical complication				
Streptozotocin (STZ)-induced diabetes during gestation	Yes	12	↔ GFR, ↑ BP, tubulointerstitial injury	[73]
Partial ligation of uterine ligation	Yes	2	↓ GFR, glomerular hypertrophy	[74]
Lipopolysaccharide (0.79 mg/kg/day) i.p. at gestational day 8, 10, and 12	Yes	7	↓ GFR	[75]
Environmental chemical exposure				
Ethanol (1 g/kg/day) at gestational day 13.5 and 14.5	Yes	4	↓ GFR at 6 mo	[76]
DEHP exposure (0.25 or 6.25 mg/kg/day) during pregnancy	Yes	21	↓ GFR, ↑ BP	[77]
Medication use				
Dexamethasone (0.1 mg/kg/day) during gestation	Yes	8	↓ GFR, glomerular hypertrophy	[78]
Dexamethasone (0.2 mg/kg/day) at gestational day 15 and 16 or 17 and 18	Yes	8	↔ GFR, unchanged glomerular morphology	[79]
Dexamethasone (0.1 mg/kg/day) from gestational day 16 to 22.	Yes	16	↑ BP	[80]
Cyclosporine (3.3 mg/kg/day) from gestational day 10 to postnatal day 7	Yes	12	↔ GFR, glomerular hypertrophy	[81]

GFR = glomerular filtration rate; DEHP = di-2-ethylhexylphthalate; ↑ = increase; ↓ = decrease; ↔ = no difference.

## Data Availability

Data are contained within the article.
